# Effect of Dietary Eicosapentaenoic and Docosahexaenoic Fatty Acid Supplementation during the Last Month of Gestation on Fatty Acid Metabolism and Oxidative Status in Charolais Cows and Calves

**DOI:** 10.3390/ani14091273

**Published:** 2024-04-24

**Authors:** Diana Brozić, Kristina Starčević, Marina Vranić, Krešimir Bošnjak, Maja Maurić Maljković, Tomislav Mašek

**Affiliations:** 1 Department of Animal Nutrition and Dietetics, Faculty of Veterinary Medicine, University of Zagreb, 10000 Zagreb, Croatia; tmasek@vef.unizg.hr; 2 Department of Chemistry and Biochemistry, Faculty of Veterinary Medicine, University of Zagreb, 10000 Zagreb, Croatia; kstarcevic@vef.unizg.hr; 3 Department of Field Crops, Forage and Grassland Production, Faculty of Agriculture, University of Zagreb, Svetošimunska Cesta 25, 10000 Zagreb, Croatia; mvranic@agr.hr (M.V.); kbosnjak@agr.hr (K.B.); 4 Department of Animal Breeding and Livestock Production, Faculty of Veterinary Medicine, University of Zagreb, 10000 Zagreb, Croatia; mmauric@vef.unizg.hr

**Keywords:** peripartal period, eicosapentaenoic acid, docosahexaenoic acid, cholesterol, lipogenesis, antioxidative status

## Abstract

**Simple Summary:**

Although dietary enrichment with n-3 fatty acids has been extensively studied in cows, there are limited data on the metabolic adaptation of n-3 long-chain polyunsaturated fatty acids during the peripartal period in cows and their calves. We found a significant effect of low-dose eicosapentaenoic acid and docosahexaenoic acid supplementation during late gestation on fatty acid metabolism in cows. Namely, the fatty acid composition of the colostrum, early milk, and plasma was significantly altered. No effect was observed in the plasma of the calves, indicating rapid utilization of the long-chain polyunsaturated fatty acids by the newborn. Furthermore, no adverse effects were observed related to n-3 fatty acid supplementation, such as reduced gene expression for de novo fatty acid synthesis, depression of milk fat, or changes in oxidative status.

**Abstract:**

Fatty acids (FAs) are of utmost importance in the peripartal period for the development of the central nervous and immune systems of the newborn. The transport of polyunsaturated fatty acids (PUFAs) through the placenta is considered to be minimal in ruminants. Nevertheless, the cow’s FAs are the main source of FAs for the calf during gestation. This research aimed to investigate the influence of low-dose eicosapentaenoic acid (EPA) and docosahexaenoic acid (DHA) supplementation during late gestation on the FA metabolism of cows and their calves. A total of 20 Charolais cows during the last month of their gestation were included in the feeding trial and were divided into a control group (CON) and an experimental group (EPA + DHA). The latter received a supplement in the amount of 100 g/day (9.1 and 7.8 g/cow/day of EPA and DHA, respectively). Supplementation of low-dose EPA and DHA alters colostrum and milk fatty acid composition through the elevation of n-3 long-chain polyunsaturated fatty acids (LC-PUFAs) without affecting milk fat and protein concentrations and oxidative status. Plasma composition in cows was significantly altered, while the same effect was not detected in calf plasma. No significant change in mRNA expression was detected for the genes fatty acid synthase (FASN) and acetyl-CoA carboxylase alpha (ACACA).

## 1. Introduction

Polyunsaturated fatty acids EPA and DHA are known to influence the development of the central nervous system and the immune system; therefore, their presence in the last trimester of gestation is crucial in all mammalian species [1,2]. To date, the effects of supplementation with LC-PUFA in the last trimester of gestation on the FA metabolism of both the cow and the calf have only been researched to a limited extent [3]. Passage of PUFAs through the bovine placenta is thought to be minimal in ruminants [4]. Nevertheless, FAs in the maternal circulation are the major source of FAs utilized by the fetus [5]. In addition, the placenta of gestating cows is thought to regulate the transfer and synthesis of LC-PUFAs to meet the requirements of newborns in a preferential and timely manner [6]. It is known that epitheliochorial placentas are less permeable to free FAs than hemochorial ones. Nevertheless, FAs are still transported via several membrane-bound carrier proteins [7,8]. However, the specific mechanisms of these proteins in placental fatty acid uptake, metabolism, and transfer are not fully known [6]. The FA composition of milk and FA synthesis in mammary gland tissue are under the direct influence of the lactation phase and nutrition [9,10,11]. Both the stage of lactation and the energy balance contribute significantly to variations in milk fat composition and alter the activity of different fatty acid pathways [12]. Diet is a dominant external factor that has a direct influence on FA synthesis and composition [13]. n-3 LC-PUFA fatty acids, namely EPA and DHA, are found in high concentrations in fish oil and algae [14]. In contrast, their concentration is negligible in milk [15]. Modifying the fatty acid composition in milk through supplementation of n-3 LC-PUFA has been frequently investigated in recent years [16,17,18,19]. The aim of altering the FA composition in milk is to increase the content of LC-PUFAs to enhance the beneficial effects of n-3 FA on human health through the consumption of fortified milk and meat [20,21]. In addition, the enrichment of milk with n-3 LC-PUFA in cows leads to a lower concentration of FAs in milk that are classified with potentially negative, atherogenic effects on human health—namely, lauric, myristic, and palmitic acid [22,23]. Such a change in the FA composition in milk can also alter the organoleptic properties by affecting the oxidative stability of the milk [24]. Furthermore, PUFA not only influences the nutritional properties of milk but also impacts lipogenesis by regulating gene expression in milk and transcriptional pathways [25]. De novo FA synthesis in the mammary gland is inhibited under the influence of certain PUFAs and their trans isomers, which are the result of rumen microbiome biohydrogenation [26].

The aim of this study was, therefore, to investigate the effects of supplementation of EPA and DHA FAs in Charolais cows during late gestation on the FA profile of colostrum and early milk and the blood plasma of cows and their calves. In addition, we studied the effects of supplementation on the chemical composition of colostrum and early milk, oxidative status in plasma and milk, and the effects on the gene expression of ACACA and FASN in the blood of cows and their calves in the postpartum period.

## 2. Materials and Methods

### 2.1. Animals and Experimental Design

The trial was conducted at the experimental facility of the Centre for Grassland Production, Sljeme, Faculty of Agriculture, University of Zagreb, and the analyses were performed at the Department of Animal Nutrition and Dietetics, Faculty of Veterinary Medicine, University of Zagreb. The experimental protocol was approved by the Ethics Committee of the Faculty of Veterinary Medicine of the University of Zagreb. A total of 20 Charolais breed cows during the last month of their gestation were included in the feeding trial. The animals were kept on deep litter bedding, separated by groups. After calving, the calves were kept together with their mothers until weaning. All cows were fed a basic diet consisting of alfalfa haylage, ground maize, and vitamin-mineral supplement. The cows were divided into a control group (CON) and an experimental group (EPA + DHA). The latter received a fish oil-based fat supplement (FATMIX 65, AND Nutrition, Spain) at the amount of 100 g/day (9.1 and 7.8 g/cow/day of EPA and DHA, respectively) for a period of 30 days before the expected calving. Water was provided ad libitum. Blood samples were collected on the day of calving from cows and their calves (*v. jugularis*). Colostrum samples were collected 6 h after calving, and milk samples were collected on the 7th day after calving. Collected samples were stored at −80 °C until further analysis. Samples of the feed were ground and analyzed according to the AOAC procedures [27] (Table 1 and Table 2). Colostrum and milk samples were analyzed for fat, protein, and lactose by infrared spectrophotometry (Fossomatic 4000 Milkoscan Analyzer, Foss North America, Brampton, ON, Canada). In serum, an analysis of the following blood biochemistry parameters was performed: glucose, triacyclglycerols, total cholesterol, HDL cholesterol, and LDL cholesterol (SABA 18, AMS, Marcianise, Italy). The calves were weighed after calving on days 0, 14, 21, and 28 on an electronic scale with a precision of 100 g.

### 2.2. Fatty Acids Analysis

The analysis of FA was performed in colostrum and milk samples as well as in blood plasma samples from cows and their calves according to a method previously described by Mašek et al. [23]. The fat was extracted using a mixture of isopropanol and hexane (3:2) according to the method described by Hara and Radin [28]. Transesterification of the FA methyl esters was carried out with a 20% solution of boron trifluoride in methanol. The analysis of FA was performed using a Shimadzu GC2010Plus gas chromatograph with a flame ionization detector and a 30-m capillary column ZB WAX (Phenomenex, Torrance, CA, USA). All experimental measurements were performed in triplicate, and the average values were reported. Quantification was performed by area normalization with an external standard mixture of fatty acid methyl esters (37 component FAME mix, PUFA No3, Sigma-Aldrich, Darmstadt, Germany). The FA composition was calculated as the percentage of each fatty acid in relation to the total FA.

### 2.3. Determination of Thiobarbituric Acid-Reactive Substances (TBARS)

Malondialdehyde (MDA) content was measured using the high-performance liquid chromatography method in the plasma of cows and their calves as described by Grotto et al. [29]. The method is based on the reaction of MDA with thiobarbituric acid (TBA), which gives a red color and forms a complex of TBARS, the concentration of which is determined. TBARS were determined with a Shimadzu 2010 HPLC (Shimadzu, Tokyo, Japan) using an InertSustain C-18 column (4.6–150 mm–5 µm) (GL Sciences Inc., Tokyo, Japan) and a UV/Vis detector. The standard curve was prepared using 1,1,3,3-tetraethoxypropane. TBARS were expressed as μM.

### 2.4. Determination of Total Phenols in Milk

Total phenols were determined spectrophotometrically by measuring the resulting color intensity at a wavelength of 725 nm. The method is based on the reaction of phenol with the Folin–Ciocalteu reagent. Samples were prepared as follows: 0.5 mL of milk, 15 mL of distilled water, and 1 mL of 10% Folin–Ciocalteu reagent were pipetted into a 25 mL volumetric flask and were mixed. Then, 0.8 mL of 7.5% Na_2_CO_3_ was added to the prepared mixture and diluted with distilled water. The sample was mixed and incubated at room temperature for 90 min. The absorbance was measured at a wavelength of 725 nm. Each sample was prepared in triplicate [30]. To calculate the concentration of total phenols, a calibration curve was created using gallic acid as a standard [31]. The concentration of total phenols was calculated and expressed in mg gallic acid equivalent (GAE)/L of milk.

### 2.5. Determination of Cholesterol in Milk

Cholesterol was determined using a modified method, previously described by Fletouris et al. [32]. A Shimadzu 2010 LC system (Shimadzu, Japan) equipped with an InertSustain C18 (4.6–150 mm–5 µm) column (GL Sciences Inc., Japan) and a UV/Vis detector were used for the analysis of cholesterol. The total cholesterol content in milk was calculated in duplicate for each milk sample (values accepted for CV < 6%), based on the external standard technique, from the standard curve of the peak area vs. concentration range.

### 2.6. Determination of FASN and ACACA Gene Expression in Whole Blood

FASN and ACACA gene expression were determined using quantitative PCR in the blood of cows and their calves. Total RNA was extracted from whole blood samples (with anticoagulants) using the acid guanidinium thiocyanate-phenol-chloroform extraction method with ONE STEP RNA Reagent (Bio Basic Inc., Markham, ON, Canada). All the preparation steps were carried out according to the manufacturer’s instructions. The quantity of isolated RNA samples was checked by spectrophotometry (BioDrop μ LITE, BioDrop, Cambridge, UK). Total RNA was subjected to a two-step RT-qPCR reaction. In the first phase, the PCR program was started with a reverse transcription reaction using PrimeScriptTM RT reagent (TaKaRa Bio Inc., Shiga, Japan) according to the manufacturer’s manual. In the second phase, the PCR reaction was performed using a Real-Time PCR reagent, SYBR^®^ Premix Ex Taq (TaKaRa Bio Inc., Shiga, Japan). The RT-qPCR was performed using a Stratagene MxPro3005 (Agilent Technologies, Mississauga, ON, Canada) thermocycler. The primers used in this study are listed in Table 3. Relative expression ratios were calculated as normalized ratios to housekeeping gene glyceraldehyde-3-phosphate dehydrogenase (GAPDH) [33]. The final relative gene expression ratios were calculated using the ΔΔ Ct method (2^ΔΔCt^) and results were expressed as fold change relative to control. The relative mRNA quantity in each sample was normalized to that of b-actin according to the equation [34]:

ΔCT = CT (a target gene) − CT (a reference gene) ΔΔCt = ΔCT (a target sample) − ΔCT (a reference sample) R = 2^ΔΔCt^

### 2.7. Statistical Analysis

Data were analyzed using GraphPadPrism10 software, comparing between the groups of cows CON vs. EPA + DHA and their calves CON vs. EPA + DHA using the unpaired *t*-test (GraphPad Software, Inc., San Diego, CA, USA). The normality of distribution was tested with the Shapiro–Wilks test. All data are presented as means ± standard deviation. The significance was set at *p* < 0.05. Standard deviations of fold changes for the tested gene expression were determined according to the previously described protocol [37].

## 3. Results

The fatty acid composition of colostrum and milk in the experimental group (EPA + DHA) was significantly altered. We found higher concentrations of fatty acids EPA, DHA, docosapentaenoic (DPA), and total n-3 LC-PUFA in colostrum, and EPA, DPA, and the total n-3 LC-PUFA in milk on the 7th day of lactation (Table 4).

**Figure 1:**
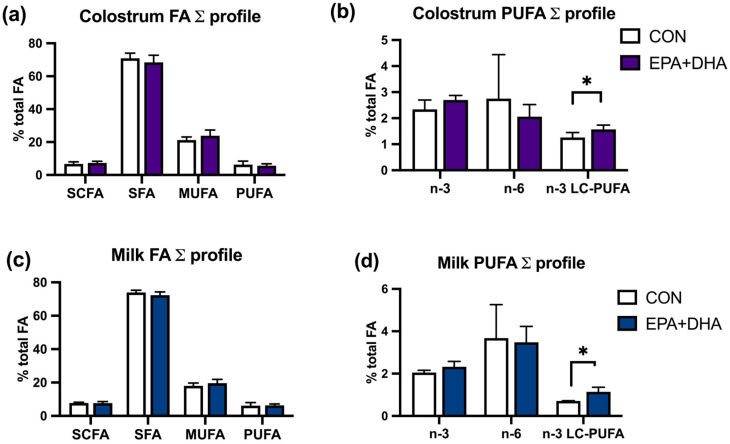
The effect of fatty acid supplementation (CON vs. EPA + DHA) in colostrum and 7th day of milk on the summed fatty acid composition of milk fat: (**a**) proportion (g/100 g of total fatty acids) of SCFAs—short-chain fatty acids (C4–C10), SFAs—saturated fatty acids; MUFAs—monounsaturated fatty acids, PUFAs—polyunsaturated fatty acids in colostrum; (**b**) and n-3 fatty acids, n-6 fatty acids, n-3 LC-PUFAs—n-3 long-chain polyunsaturated fatty acids in colostrum; (**c**) proportion (g/100 g of total fatty acids) of SCFAs, SFAs, MUFAs, PUFAs on 7th day of milk (**d**) and n-3, n-6, n-3 LC-PUFAs of 7th day of milk. CON—control group. EPA + DHA—experimental group. * *p* < 0.05. *n* = 10/group.

Nevertheless, the summed profile of saturated fatty acids (SFAs), monounsaturated fatty acids (MUFAs), and PUFAs was not altered (Figure 1). The milk fat and protein concentration in colostrum and milk did not differ between groups (Table 5).

**Figure 2:**
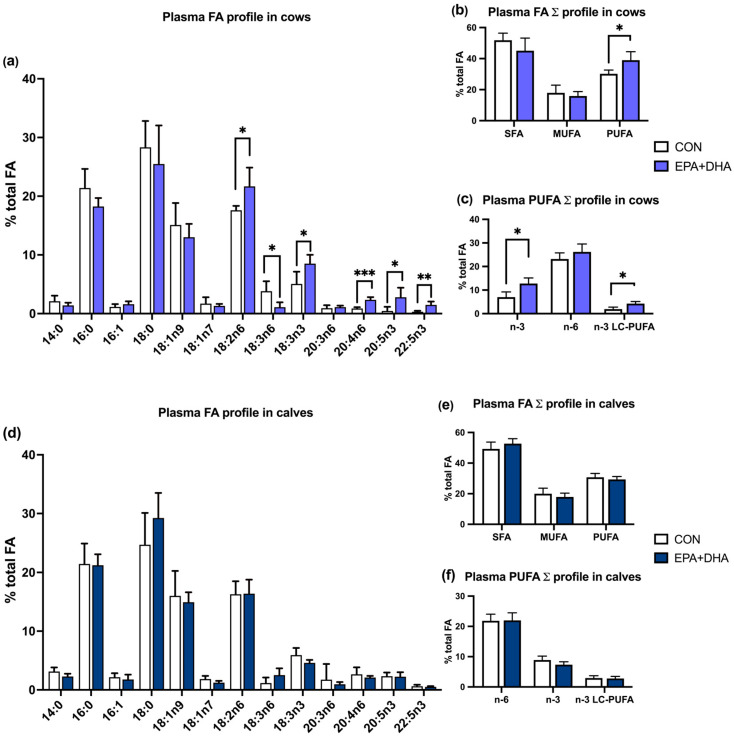
The effect of fatty acid supplementation (CON vs. EPA + DHA) in plasma of cows and their calves at calving on the fatty acid composition of milk fat: (**a**) proportion of fatty acids (g/100 g of total fatty acids) in cow plasma; (**b**) proportion of summed profile of fatty acids (g/100 g of total fatty acids) in cow plasma: SCFAs—short-chain fatty acids (C4–C10), SFAs—saturated fatty acids; MUFAs—monounsaturated fatty acids, PUFAs—polyunsaturated fatty acids in cow plasma; (**c**) proportion of summed profile of fatty acids (g/100 g of total fatty acids) n-3—omega 3 fatty acids, n-6—omega 6 fatty acids, n-3 LC-PUFAs—omega 3 long-chain polyunsaturated fatty acids in cow plasma; (**d**) proportion (g/100 g of total fatty acids) of fatty acids in calves plasma; (**e**) proportion (g/100 g of total fatty acids) of SCFAs, SFAs, MUFAs, PUFAs in calves plasma; (**f**) and n-3, n-6, n-3 LC-PUFAs in calves plasma. CON—control group. EPA + DHA—experimental group. Significance was set at * *p* < 0.05; ** *p* < 0.01; *** *p* < 0.001. *n* = 10/group.

In cow plasma, concentrations of the summed profile of PUFAs, n-3 FAs, and n-3 LC-PUFAs were significantly higher in the experimental (DHA + EPA) group. The plasma FA composition of calves was not affected by the supplementation (Figure 2).

**Figure 3:**
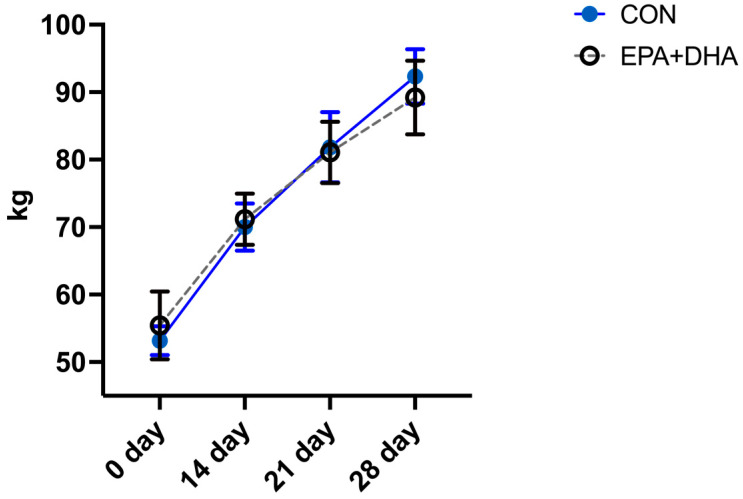
The effect of fatty acid supplementation (CON vs. EPA + DHA) in cows on the body weight of their calves in the postnatal period. CON—control group. EPA + DHA—experimental group. Significance was set at *p* < 0.05. *n* = 10/group.

**Figure 4:**
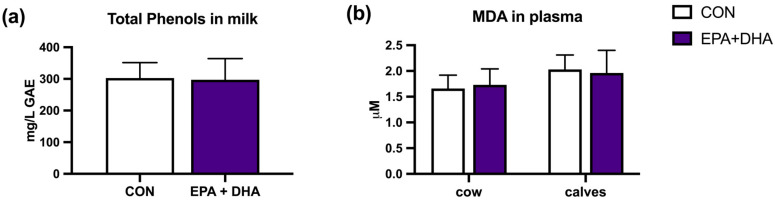
The effect of fatty acid supplementation (CON vs. EPA + DHA) on oxidative status: (**a**) Gallic acid equivalent (GAE) in 7th-day milk; (**b**) Malondialdehyde (MDA) in plasma of cow and calves. CON—control group. EPA + DHA—experimental group. Significance was set at *p* < 0.05.

**Figure 5:**
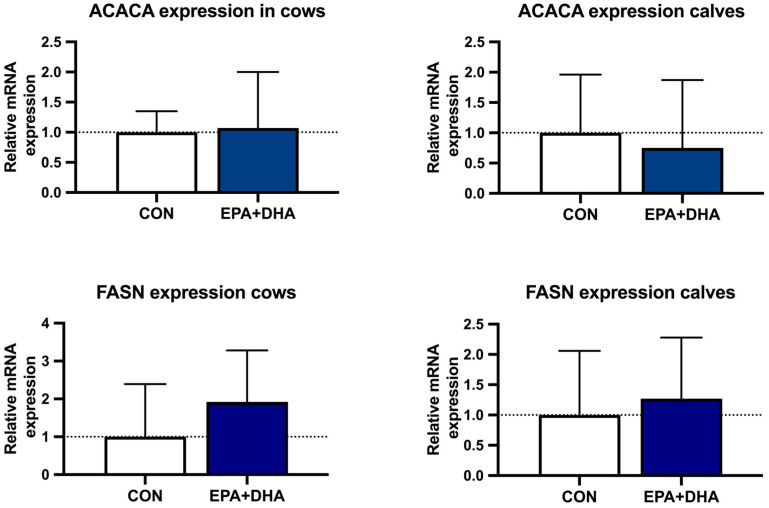
The effect of fatty acid supplementation (CON vs. EPA + DHA) on ACACA and FASN gene expression in the blood of cows and their calves; CON—control group; EPA + DHA—experimental group; significance was set at *p* < 0.05. *n* = 10/group.

The biochemistry serum lipid profile of cows and their calves was not altered by the supplementation protocol, other than total cholesterol concentration, which was significantly lower in the EPA + DHA group of cows (Table 6). The body weight of calves also did not differ between groups (Figure 3). Oxidative stress markers in plasma, MDA concentration, and, in milk, the concentration of total phenolic compounds, showed no difference between groups, in both cows and calves (Figure 4). Relative mRNA abundance for genes FASN and ACACA in cow and calf blood was not affected by the treatment (Figure 5).

## 4. Discussion

Over the last two decades, research has focused on modifying the fatty acid composition of animal-based products to improve properties that could be beneficial for human health. At the same time, just a few studies have aimed to determine whether it is possible to influence lipid metabolism, which may benefit the animal itself. In this way, a diet supplemented with n-3 PUFAs could serve as a nutraceutical for both the animals and the end consumers of enriched animal products. In addition, the role of LC-PUFAs in the developmental programming of ruminants has received significant interest, and there is evidence that FA supplementation may be a viable alternative to optimize offspring health and subsequent productivity [6]. However, ruminal biohydrogenation (RBH) not only alters the PUFA concentration in milk but also significantly reduces its concentration. The presumed percentage of rumen biohydrogenation of unprotected unsaturated FAs (UFAs) exceeds 80% [38]; for EPA and DHA, it is estimated to be 78–100% and 74–98%, respectively [39,40]. Milk is naturally a poor source of EPA and DHA, estimated to be less than 0.1% of total FAs [15,41]. However, the inclusion of fish oils and algae in the diet has been shown to increase the level of n-3 LC-PUFA in milk, particularly EPA and DHA [41,42].

Nevertheless, no consistent results were obtained for the transfer from feed to milk, as the characteristics of the supplementation and the concentration of n-3 PUFAs in the diet influence this percentage [43]. The efficiency of transfer from feed to milk is estimated to be about 2.6% for EPA and 4.1% for DHA [24]. These results were confirmed in our study, in which we found that the fatty acid composition of colostrum in the experimental group (DHA + EPA) was significantly altered after supplementation with a low-concentration supplement of n-3 PUFA. We found higher concentrations of the fatty acids EPA, DHA, docosapentaenoic acid, oleic acid, and stearic acid. The summed profile of saturated fatty acids (SFAs), monounsaturated fatty acids (MUFAs), and PUFAs was not altered, but the summed profile of n-3 LC-PUFAs was significantly higher in the EPA + DHA group. These results indicate that fat supplements high in DHA and EPA modified the fatty acid profile of colostrum milk fat and increased the concentration of FAs beneficial for calf health in the perinatal period. Feeding oil supplements to dairy cows can also reduce the milk fat content of unfavorable saturated FAs (SFAs), including lauric, myristic, and palmitic acid [23]. In our study, the addition of fat supplementation significantly reduced the concentration of myristic acid in colostrum. This effect is mainly attributed to the down-regulation of mammary lipogenic gene expression [44,45].

In previous studies, n-3 PUFA supplementation was found to have a negative effect on milk fat concentration, leading to the milk fat depression (MFD) effect [46]. Fish oil supplementation at high concentrations in lactating cows can lead to a decrease in milk fat [40], which can ultimately lead to a slower growth rate of the offspring [47]. Therefore, the optimal concentration and duration of supplementation were achieved in our study, which ultimately did not result in MDF in the experimental group. Moreover, our results show a higher SFA concentration in colostrum for caproic (C6:0) and caprylic fatty acids (C8:0), which indicated that de novo FA synthesis was not affected at a significant level [48]. Previous studies have found that supplementation with a high PUFA content decreases all de novo synthesized FAs in milk, with the effect being more pronounced for C8:0 to C16:0 compared to C4:0 and C6:0 [49]. The body weight of the calves also did not differ between the two groups during the 28-day post-calving period. Meanwhile, the residual effect of EPA and DHA supplementation, confirmed by the increase in n-3 PUFA concentration in milk 7 days after calving, indicates that the calves in the experimental group ingested higher concentrations of n-3 LC-PUFAs, which may consequently have a positive effect on the immune system response. However, the increase in colostrum found in our study is lower than the FA inclusion provided in the study by Ballou et al. (0.83 for EPA and 0.15 for DHA compared to 1.3 for EPA and 1.1 for DHA g/100 g FA) [50], where beneficial effects on the immune response were found. Therefore, further studies are warranted to investigate the effect of low-dose EPA + DHA supplementation at 0.09% DM in the cow’s diet during gestation on calf immunity. PUFAs, namely DHA and EPA, are crucial for the development of the nervous system in the neonatal period [51]. Their importance is essential with regard to both neonatal mortality [47] and neonatal vitality and vigor [52,53]. The FA composition is influenced by supplementation not only in milk fat but also in plasma phospholipids [54]. Our results confirmed earlier findings by Childs et al. [55], namely their results showcased significantly higher concentrations of n-3 PUFA in the plasma of cows. These results are consistent with our findings showing that EPA and DHA supplementation in the last trimester of gestation at low concentrations has a significant effect on the FA composition of cows’ plasma. Nevertheless, those effects were not found in the plasma of calves. Likewise, we found significantly higher concentrations of EPA and DPA in the plasma of EPA + DHA cows, but the effect did not translate to the FA composition in calf plasma. The reason for these results is presumably the limited transplacental transport of PUFAs in the ruminant [56]. The regulation of transplacental transport is still a scientific field in which there are many unanswered questions. Previous studies have shown that there is a strict regulation of transplacental transport in favor of DHA, an essential fatty acid for the development of the nervous system [3]. During gestation, n-3 LC-PUFA is incorporated into the membrane phospholipids of nervous system tissues and other structures. The neonatal period is a period of active immunization of calves and their first contact with antigens. Therefore, any beneficial effects of n-3 PUFAs on the immune system could also translate to their effects later in life [50]. Moallem and Zachut [57] found that the fatty acid composition in the plasma of cows and calves on the first day after parturition after they had been supplemented with encapsulated fish oil (EPA and DHA provided at 5.8 and 4.3 g per cow/day, respectively) during gestation was altered significantly, and plasma DHA concentration increased by 1.9 times. Although a lower level of supplementation was used, these results differ from ours. The reason for this could be the supplementation of SFAs in the control group, as opposed to no control group supplementation in our study, and a high concentration of ALA in the basal diet. In our study, the levels of DHA in the plasma of calves were similar in both groups compared to the studies where differences in the plasma of calves were found between treatments (0.5% of FAs in the study by Moallem and Zachut and 0.57% of FAs in the study by Marques et al. in groups supplemented with fish oil versus 0.65% of FAs for EPA + DHA and 0.52% of FAs for the CON group in our study) [57,58]. Moreover, the FAs used in our study, EPA and DHA, had a higher degree of desaturation compared to ALA, and therefore their bioavailability for implementation in developing tissues, namely neurological structures and biological membranes, is thought to be greater [59,60]. Similar results, i.e., no significant changes in the plasma of the offspring, were found in a study in which sheep were supplemented with a concentration of EPA + DHA during gestation that was 2.5-fold lower compared to our study [61]. The authors conclude that the administered dose was insufficient to modify ewe and lamb metabolic status and performance through weaning [62]. Nevertheless, we found positive effects on FA concentrations in colostrum, early milk, and cow plasma. Therefore, an analysis of FAs in additional tissues would shed light on whether the lack of change in FAs in plasma is due to rapid FA utilization or whether the amount of supplementation used here was too low to show significant changes in calf plasma.

In addition to a positive increase in LC-PUFAs in the plasma of calves after birth, a study by Uken et al. also pointed to the potentially negative effects of supplementing the dam’s diet with a high-concentration oil supplement (linseed oil, rich in ALA, at a rate of 40 g/cow per day during gestation) [63]. They found a reduced milk fat content in colostrum even though FAs were administered directly into the abomasum. The biohydrogenation of PUFAs alongside the formation of trans-FAs in the rumen was avoided, which was previously considered to be the main cause of MFD [57]. In addition, a decrease in de novo FA synthesis in the mammary gland was observed [64].

We have found that supplementation with low-dose EPA and DHA enables a sparing effect for essential fatty acids, as evidenced by higher concentrations of these PUFAs in plasma—α-linolenic acid (ALA) and linoleic acid (LA). Other LC-PUFAs are also visible in the EPA + DHA cow plasma. Meanwhile, the same effect was not detected in calf plasma, which suggests the fast utilization of n-3 LC PUFAs through tissue incorporation during the last month of gestation. A higher concentration of essential FAs, ALA, and LA is beneficial as it can serve as a pool for FA bioconversion for LC-PUFA synthesis. The sparing effect detected in the EPA + DHA group of LC-PUFAs suggests that the desaturation rate was increased in the CON group of cows, leading to a leveling of plasma concentrations of LCPUFAs in the calves due to their essential nature during the peripartum period. In addition, the fatty acid profile of the CON and EPA + DHA diets differed significantly in terms of EPA and DHA (Table 2). Nevertheless, the ALA concentration was relatively high in the basal diet (0.7% on a DM basis versus 0.14% in the study by Uken et al. [63]) due to the high inclusion of alfalfa haylage in the diets (approximately 10 kg on a DM basis per cow/day). Therefore, we can hypothesize that the calves in the control group received a high amount of ALA during fetal development, which provides the pool for desaturation and elongation to EPA and DHA and has ultimately resulted in similar concentrations of LCPUFAs in calves’ plasma. The bioconversion to LC-PUFAs, EPA, and DHA is upregulated in the fetus due to the high demand during fetal growth [59,65]. Additionally, placental lipase activity increases during the final trimester of pregnancy, enhancing placental fatty acid delivery to the fetus [7].

The biochemical profiles (glucose, total cholesterol, HDL cholesterol, LDL cholesterol, and TAG) of the cows did not differ between the groups, except for cholesterol concentration, which was lower in the experimental group. Research has shown [66] that long-chain n-3 PUFAs can improve blood lipid profiles by decreasing serum triglycerides and increasing HDL cholesterol concentration and LDL particle size. Nevertheless, the only change we noted was a decrease in total cholesterol in the supplemented cow group, and the effect did not translate to the calf lipid blood profile.

The concentration of MDA, a marker for oxidative stress, in cow and calf plasma did not differ between the groups. This leads to the conclusion that the oxidative status in plasma remained unaffected. The suspected reason for this phenomenon is a low concentration of EPA + DHA supplementation in contrast to the studies by Wullepit et al. [67], where cows were fed 44 g DHA per day 3 weeks before calving. Cows included in the treatment showed significantly higher TBARS concentrations in blood plasma than the cows in the control group. In addition, a study by Scislowski et al. [68] has shown that supplementation with linseed oil in cows leads to a similar effect. Likewise, the oxidation of LC-PUFAs in milk leads to the formation of free radicals that affect the quality of the milk; therefore, the addition of LC-PUFAs may reduce the antioxidative stability of the milk. Peroxidation is triggered by cellular ROS, in which hydroxyl, alkoxyl, or peroxyl radicals split off hydrogen from a PUFA acyl group [69], suggesting that an increase in PUFA levels may contribute to a decrease in antioxidant capacity and an increase in lipid peroxidation products [70]. Antioxidants, including dietary tocopherols and carotenoids, slow down the process of lipid peroxidation and the formation of free radicals [71,72]. Forages are a natural source of antioxidants and can serve as a protective factor against oxidation. In a study conducted by Glover et al. [19], where a protected algae oil supplement was used when measuring the oxidative stability and antioxidant capacity of the milk, it was found that there were no differences between the experimental and control groups. This is consistent with our findings, as we found no change in the concentration of total phenols between the milk of the control and experimental groups. Studies in which the sensory properties of milk were assessed after the addition of EPA and DHA to the diet also showed no negative effect of the n-3 LC-PUFA supplementation [73,74,75].

MFD is a phenomenon in which milk fat yield is significantly reduced with no change in milk yield and is typically observed in cows that are fed diets supplemented with plant or marine lipid sources [26]. Previous studies have established reduced milk fat synthesis in cows supplemented with fish oil due to the decreased gene expression of mammary lipogenic enzymes, ACACA, FASN, and Stearoyl-CoA desaturase [76,77,78,79]. The extent of the reduction in milk fat content is directly correlated to the amount of PUFA supplementation [80]. Another proposed mechanism is that feeding marine oils leads to increased ruminal outflow of specific RBH intermediates, which may subsequently act as inhibitors of lipogenesis in the mammary gland, leading to MFD. This suggests that, in our study, the amount of EPA and DHA supplemented was not high enough to cause MFD but resulted in positive changes in the FA composition of the colostrum and early milk.

Acetyl-CoA and butyryl-CoA are the major precursors for de novo fatty acid synthesis in ruminants and are formed from acetate and beta-hydroxybutyrate (BHBA) [81]. The de novo FA synthesis pathway is regulated by the activity of two enzymes, ACACA and FASN. ACACA catalyzes the synthesis of malonyl-CoA from acetyl-CoA, and the FASN enzyme catalyzes the elongation of either a primary malonyl-CoA or butyryl-CoA [82]. Here, we investigated the interaction of EPA and DHA supplementation and the expression of the ACACA and FASN genes involved in lipogenesis in the blood of cows and their calves. PUFAs, including EPA and DHA, not only alter the nutritional properties of milk but also influence gene expression [25]. It is known that the de novo synthesis of milk fat in the mammary gland involves the coordinated expression of multiple regulators of transcription and their target genes [83]. The regulatory mechanisms are largely adapted to the altered diet in the initial phase of treatment; therefore, the differences in the expression of individual genes and transcription factors also depend on the duration of treatment [22,44,76,77]. The FA profile of milk and fat synthesis in the mammary gland is directly influenced by the stage of lactation and the composition of the diet [9,10,11]. However, diet is a dominant external factor that has a direct effect on milk fat synthesis. Previous research has shown that the de novo synthesis of FAs in the mammary gland is inhibited by certain FAs and their trans isomers formed through biohydrogenation by the microflora in the rumen [26]. Conjugated linoleic acid (CLA) is considered to inhibit de novo FA synthesis [84]. In the case of the MDF phenomenon, the transcription of genes involved in the de novo synthesis of milk fat is also reduced [26]. The molecular mechanisms involved in the regulation of the inhibition of de novo synthesis are not fully understood to date, and the role of the transcription factors SREBP in regulating this pathway has been proposed as a universal regulator of the expression of many genes involved in fat synthesis [46,85,86]. This was confirmed by studies on cell cultures of the mammary gland of a cow. It was also found that trans 10, cis-12 CLA influences the reduced expression of the SREBP1 protein in the cells [87]. In addition to their role as energy molecules, FAs also play the role of signaling molecules involved in the regulation of gene expression [88]. FAS has been found to be regulated via a negative feedback pathway by PUFAs, which reduce the expression of the gene SREBP 1 and thus have a negative effect on the transcription of the FASN gene [89]. Kadegowda et al. [90] found that the addition of LC-PUFAs to the cow’s diet alters the regulation of the de novo synthesis of short- and medium-chain fatty acids as well as the mRNA expression of enzymes involved in de novo synthesis in the cow’s mammary gland tissue [91]. De novo synthesis of fatty acids is most active during the first month of lactation [83]. Therefore, the lack of reduced mRNA expression of the ACACA and FASN genes in the blood of the cows in our experimental group is not surprising, as the level of n-3 LC-PUFA supplementation was low. It was also assessed on the first day of lactation, therefore the reduced expression seems to be counteracted by protective mechanisms for de novo fatty acid synthesis. Additionally, since the expression in our study was not determined in the mammary gland tissue but in the whole blood of the cows, we can speculate that more specific results would be derived when assessing gene expression in the mammary tissue or liver tissue, considering previous results that suggest a tissue-specific regulation of genes coding for lipogenesis enzymes [11,92,93]. Similar to our results, the study of Murrieta et al. [94] found no attenuating effect on the mRNA expression of ACACA and FASN in the somatic cells of the mammary gland after the ingestion of a diet with high concentrations of LA-rich sunflower oil.

## 5. Conclusions

We conclude that supplementation with low-dose fatty acids EPA and DHA alters the composition of colostrum and milk fatty acids by increasing n-3 LC-PUFAs without affecting milk fat and protein concentration and oxidative stability. Plasma composition in cows was significantly altered during supplementation, while the same effect was not observed in calf plasma. Supplementation with low-dose EPA and DHA during gestation alters plasma composition in cows and enables a pool for incorporation in milk and peripheral tissues. The observed sparing effect of essential fatty acids ALA, LA, and other LC-PUFAs is visible in the EPA + DHA group of cows. Meanwhile, the same effect was not detected in calf plasma, which suggests fast LC-PUFA utilization during the last month of gestation alongside upregulated desaturation of essential fatty acids.

In blood, no significant change in the mRNA expression of the ACACA and FASN genes was detected, suggesting that there is a tissue-specific mechanism regulating the expression of lipogenic genes and that the expression of the genes is dose-dependent. An in-depth study analyzing the mammary gland, endometrium, and liver tissue, alongside a higher number of de novo FA synthesis biomarkers, would elucidate correlations between the de novo synthesis of FAs and FA metabolism in the peripartum period.

Low-dose EPA and DHA supplementation during the last phase of gestation shows promising results in terms of the enrichment of milk and providing a higher concentration of LC-PUFAs for suckling calves without any detrimental effects on milk fat concentration, calf growth, or oxidative stability.

## Figures and Tables

**Table 1:** Chemical composition of the diet.

% on As-Fed Basis		Alfalfa Haylage	Corn, Ground	Fatmix-65
DM		51.2	88	95
Ash		4.1	1.3	30
Ether extract (EE)		1.5	3.7	65
Crude protein (CP)		9.4	8.1	/
Crude fiber (CF)		15.4	2.7	/
Neutral detergent fiber (NDF)		22.2	12.8	/
Acid detergent fiber (ADF)		7.1	3.5	/
NSC ^1^		20.8	72.2	/
EPA (g/100 g)		0.00	0.00	9.1
DHA (g/100 g)		0.00	0.00	7.8
estimated daily intake/cow	CON	ad libitum	1 kg	/
	EPA + DHA	ad libitum	1 kg	100 g

^1^ Nonstructural carbohydrates (NSC) = DM − (CP + EE + ash + NDF); CON—control group; EPA + DHA—experimental group; Fatmix-65—fish oil-based fat supplement (AND Nutrition, Spain); DM—dry matter.

**Table 2:** Fatty acid profile of the diets.

% on DM Basis	CON	EPA + DHA
Palmitic acid (16:00)	0.709	0.873
Palmitoleic acid (16:1)	0.011	0.040
Stearic acid (18:00)	0.088	0.109
Oleic acid (18:1)	0.169	0.299
Linoleic acid (18:2n6)	0.737	0.773
Linolenic acid (18:3n3)	1.139	1.140
EPA (20:5n-3)	0.000	0.083
DHA (22:6n3)	0.000	0.071

CON—control group; EPA + DHA—experimental group; DM—dry matter.

**Table 3:** Sequence oligonucleotide primers for genes ACACA, FASN, and GADPH.

Gene	Primers	Reference
ACACA	F: (5′–CAT CTT GTC CGA AAC GTC GAT–3′)	[35]
	R: (5′–CCC TTC GAA CAT ACA CCT CCA–3′)	
FASN	F: (5′–CTA CCA AGC CAG GCA GGT C–3′)	[36]
	R: (5′–GCC ATT GTA CTT GGG CTT GT–3′)	
GADPH	F: (5′–CGT GTC TGT TGT GGA TCT GAC CTG–3′)	[36]
	R: (5′–CAA CCT GGT CCT CAG TGT AGC CT–3′)	

ACACA—acetyl-CoA carboxylase alpha; FASN—fatty acid synthase; GADPH—glyceraldehyde-3-phosphate dehydrogenase; F—forward; R—reverse.

**Table 4:** The effect of fatty acid supplementation (CON vs. EPA + DHA) on colostrum and the 7th day of milk on the fatty acid composition of milk fat.

FA (% of Total FA)	Colostrum					7th Day in Milk				
	CON		EPA + DHA		*p*-Value	CON		EPA + DHA		*p*-Value
	Mean	SD	Mean	SD		Mean	SD	Mean	SD	
C4:0	4.16	1.50	3.94	1.00	0.778	1.66	0.28	1.83	0.29	0.437
C6:0	0.84	0.27	1.21	0.23	0.036	1.83	0.11	1.89	0.19	0.614
C8:0	0.50	0.11	0.70	0.15	0.043	1.29	0.09	1.30	0.18	0.929
C10:0	1.21	0.19	1.48	0.26	0.094	2.95	0.31	2.68	0.34	0.309
C11:0	0.04	0.02	0.03	0.04	0.679	0.05	0.01	0.04	0.01	0.267
C12:0	2.44	0.18	2.33	0.30	0.495	3.57	0.38	2.86	0.37	0.056
C14:0	13.22	1.12	11.44	1.22	0.036	1.46	0.27	10.79	0.68	0.104
C14:1	0.99	0.50	0.96	0.89	0.957	0.71	0.11	0.52	0.13	0.075
C15:0	1.33	0.28	1.30	0.22	0.849	1.74	0.50	1.80	0.16	0.860
C15:1	0.02	0.02	0.02	0.02	0.824	0.05	0.06	0.04	0.03	0.881
C16:0	38.98	2.80	34.57	3.78	0.066	37.47	1.44	33.97	2.65	0.052
C16:1	4.17	2.54	3.46	2.42	0.648	2.09	0.42	1.71	0.26	0.251
C17:0	0.77	0.17	0.90	0.23	0.347	0.76	0.09	0.84	0.12	0.304
C17:1	0.28	0.14	0.27	0.07	0.977	0.19	0.04	0.31	0.36	0.513
C18:0	7.20	1.80	10.21	2.24	0.043	10.86	1.30	13.71	1.62	0.040
C18:1n9	15.63	2.66	19.08	0.74	0.023	14.74	1.24	16.76	2.32	0.159
C18:2n6	2.29	1.71	1.58	0.40	0.364	3.40	1.55	3.15	0.77	0.812
C18:3n6	0.09	0.07	0.05	0.06	0.407	0.08	0.06	0.12	0.11	0.613
C18:3n3	0.92	0.18	0.90	0.14	0.876	0.86	0.06	0.79	0.10	0.268
C18:4n3	0.12	0.16	0.18	0.15	0.579	0.36	0.08	0.29	0.16	0.416
C20:0	0.16	0.04	0.21	0.08	0.262	0.26	0.04	0.43	0.29	0.257
C20:1	0.04	0.01	0.05	0.01	0.240	0.10	0.09	0.07	0.06	0.605
C20:2	0.11	0.11	0.13	0.24	0.877	0.16	0.25	0.05	0.05	0.497
C20:4n6	0.37	0.04	0.42	0.10	0.296	0.20	0.01	0.21	0.03	0.396
C20:3n3	0.04	0.03	0.05	0.03	0.820	0.11	0.13	0.10	0.09	0.919
C20:4n3	0.22	0.06	0.19	0.08	0.643	0.09	0.02	0.09	0.03	0.955
C20:5n3	0.31	0.03	0.40	0.04	0.003	0.40	0.04	0.76	0.19	0.012
C22:0	0.04	0.03	0.05	0.04	0.532	0.07	0.01	0.06	0.01	0.475
C22:1	0.02	0.03	0.01	0.01	0.447	0.03	0.01	0.03	0.03	0.757
C22:2	1.25	1.30	0.84	0.85	0.543	0.40	0.21	0.53	0.23	0.457
C22:5n3	0.61	0.11	0.83	0.08	0.005	0.18	0.01	0.22	0.03	0.038
C24:0	0.02	0.03	0.04	0.03	0.366	0.04	0.03	0.12	0.14	0.238
C22:6n3	0.12	0.01	0.15	0.01	0.001	0.04	0.04	0.07	0.06	0.428
C24:1	0.05	0.02	0.04	0.04	0.577	0.08	0.06	0.18	0.10	0.128

FA—fatty acids; CON—control group. EPA + DHA—experimental group; significance was set at *p* < 0.05. *n* = 10/group.

**Table 5:** The effect of fatty acid supplementation (CON vs. EPA + DHA) on the chemical composition of colostrum and 7th-day milk.

	%	CON		EPA + DHA		*p*-Value
		Mean	SD	Mean	SD	
Colostrum	Milk fat	4.18	1.38	3.94	2.71	0.40
	Protein	18.10	3.62	16.54	1.91	0.78
	Lactose	2.20	0.14	2.70	0.19	0.05
	Dry matter	26.03	2.42	24.67	4.17	0.35
	Fat free dry matter	21.27	3.55	20.24	1.91	0.69
	Cholesterol (μg/100 mL)	67.46	32.03	41.61	18.39	0.08
7th-day in milk	Milk fat	3.22	0.37	3.16	1.31	0.92
	Protein	3.49	0.12	3.77	0.67	0.36
	Lactose	4.76	0.17	4.61	0.26	0.34
	Dry matter	11.47	0.54	11.56	1.42	0.90
	Fat free dry matter	9.25	0.30	9.37	0.59	0.71
	Cholesterol (μg/100 mL)	10.67	2.021	12.83	4.875	0.49

CON—control group; EPA + DHA—experimental group. Results are expressed as mean ± standard deviation. *p* < 0.05. *n* = 10/group.

**Table 6:** The effect of fatty acid supplementation (CON vs. EPA + DHA) on biochemistry lipid plasma profile in cows and their calves at birth.

	Mmol/L	CON		EPA + DHA		*p*-Value
		Mean	SD	Mean	SD	
Cows	Glucose	3.28	0.33	3.03	0.39	0.258
	Total Cholesterol	3.21 ^a^	0.24	2.90 ^b^	0.14	0.025
	HDL Cholesterol	2.14	0.51	1.93	0.18	0.388
	LDL Cholesterol	1.24	0.36	1.05	0.14	0.269
	Triacylglycerol	0.15	0.06	0.14	0.04	0.687
Calves	Glucose	6.47	0.35	6.70	0.57	0.457
	Total Cholesterol	2.88	0.28	2.57	0.40	0.209
	HDL Cholesterol	1.95	0.52	1.58	0.33	0.347
	LDL Cholesterol	1.00	0.10	0.98	0.19	0.829
	Triacylglycerol	0.43	0.29	0.49	0.32	0.767

CON—control group; EPA + DHA—experimental group. Results expressed as mean ± standard deviation. Different superscript letters indicate statistical differences (*p* < 0.05) between groups. *n* = 10/group.

## Data Availability

The original contributions presented in the study are included in the article. Further inquiries can be directed to the corresponding author.
